# Let it go: How trusted reminders alter intention maintenance

**DOI:** 10.3758/s13423-026-02985-6

**Published:** 2026-08-18

**Authors:** Connor Dupre, B. Hunter Ball

**Affiliations:** Department of Psychology, University of Texas at Arlington, 501 Nedderman Drive, Arlington, TX 76109 USA

**Keywords:** Prospective memory, Cognitive offloading, Mind wandering, Distributed cognition

## Abstract

Do prospective memory (PM) reminders support intentions in a duplicative manner that preserves internal maintenance, or in a substitutive manner that reduces internal maintenance when a trusted external store is available? We tested this in a student population (*N* = 320) by crossing PM reminder reliability (0%, 50%, 100%) and availability (present vs. absent). Thought probes served as a real-time proxy for whether the intention remained in conscious awareness during the retention interval between encoding and retrieval. In Session 1 (trust trials), reminders enhanced performance without reducing PM-related thoughts, consistent with duplicative support. However, after experiencing reliable external support, Session 2 (critical trials) showed that those in the 100% condition reduced PM-related thoughts, redistributed attention to the ongoing task, and showed reduced PM retrieval when reminders were unexpectedly withdrawn. These findings suggest that trusted reminders may influence how intentions are actively maintained and prioritized during the retention interval, providing a lens for observing how memory adapts to changing external support.

**Supplementary Information:**

The online version contains supplementary material available at https://doi.org/10.3758/s13423-026-02985-6.

## Introduction

Prospective memory (PM) is the ability to remember to execute delayed intentions and is essential for everyday functioning. It enables behaviors such as taking medication, attending appointments, or sending timely messages (McDaniel & Einstein, 2007). During the retention interval between intention formation and execution, thoughts often drift toward upcoming goals. This prospective bias is well documented and may be adaptive, insofar as it reinforces intention representations and increases the probability of timely execution (Goschke & Kuhl, 1993; Kvavilashvili & Rummel, 2020; Marsh et al., 1998). However, maintaining intentions over time can impair ongoing task performance (Smith, 2003), and ruminative future-oriented thought can become cognitively burdensome (Beaty et al., 2019; Miranda et al., 2017, 2023).

One strategy to reduce this burden is *intention offloading*, or the externalization of intentions onto supports such as calendars, notes, or smartphone reminders (Risko & Gilbert, 2016). Offloading reliably improves goal fulfilment (see Gilbert et al., 2023, for a review) and reduces demands associated with effortful internal encoding (e.g., elaboration) and sustained memory maintenance (e.g., rehearsal) across delays (Risko & Gilbert, 2016; Risko et al., 2024). These benefits, however, introduce a critical theoretical question: once an intention has been externalized, how is it represented and maintained internally during the retention interval?

### Duplicative and substitutive offloading

Risko et al. (2024) propose that the downstream cognitive effects of offloading depend on how an external store is used. Individuals may attempt to store information internally even when an external system is available, referred to as *duplicative* offloading (internal + external memory representations). Under duplicative offloading, internal maintenance is preserved alongside external support, limiting the redistribution of processing capacity. Alternatively, individuals may forgo effortful internal storage and rely primarily on the external store, referred to as *substitutive* offloading (external representation only). When an external store is used in a substitutive manner, effort devoted to internal encoding and maintenance can be reduced, thereby freeing capacity that may either be redistributed to other ongoing processes or conserved. By analogy, duplicative offloading resembles backing up a file to the cloud while retaining a local copy, whereas substitutive offloading resembles transferring the file entirely to the cloud to free local resources. The choice between these strategies may be, in part, shaped by the perceived reliability of the external store and by the extent to which reliance on external storage conserves attentional resources.

### Evidence from retrospective memory

Evidence for substitutive offloading comes from retrospective memory paradigms that manipulate expectations about external memory support before encoding (Kelly & Risko, 2019a, 2019b). In these tasks, participants study words while writing each down onto a piece of paper that can later be used at test to help recall the words. When access to an external store is provided, memory performance is typically higher than in conditions without, indicating reliance on external representation. However, this benefit alone does not distinguish whether internal representations are also maintained in parallel. The distinction becomes apparent when expectations formed at encoding are violated at retrieval. Participants who are told during encoding that an external store would be available showed poorer unaided memory when access was later withheld than participants who were told from the outset that no external support would be available (Lu et al., 2020, 2022; Park et al., 2022). Risko and colleagues (2024) interpret this pattern as evidence that reliable external stores promote substitutive use by reducing reliance on effortful encoding. In contrast, expected unavailability promotes duplicative use, with internal representations maintained alongside the external store.

Retrospective memory paradigms further indicate that reliance on external stores is sensitive to perceived reliability. When the external store is believed to be trustworthy, people are more likely to reduce internal investment. But when its reliability is questioned, they preserve internal representations alongside the store (Risko et al., 2019; Pereira et al., 2022).

Converging evidence similarly shows that when external stores are perceived as unreliable or error-prone, individuals are less willing to depend on them exclusively (Runge et al., 2019; Schooler & Storm, 2021; Storm & Stone, 2015). Related findings show that when external stores are perceived as unreliable or error-prone, individuals are less willing to rely on them exclusively and instead maintain internal representations in parallel (Runge et al., 2019; Schooler & Storm, 2021; Storm & Stone, 2015).

### Intention offloading

While the findings in the retrospective memory domain support the duplicative-substitutive framework, there is reason to question whether similar findings might apply in the prospective domain. Theoretical accounts of goal activation and intention persistence would predict that individuals maintain intentions internally, regardless of external support. The intention superiority effect (Goschke & Kuhl, 1993; Marsh et al., 1998) demonstrates that intentions, once formed, are more accessible in memory than other types of information. For example, Marsh et al. (1998) found that participants were faster to recognize intention-related words during the retention interval. Likewise, the Zeigarnik effect suggests that incomplete goals are preferentially retained and tend to intrude into thought until resolved (Zeigarnik, 1927). These phenomena are central to a mechanism of intention retrieval called “prospective retrieval mode.” Rooted in Tulving’s (1983) concept of ecphory, it refers to a sustained goal-directed attentional state reflecting readiness to detect and act on intentions when the time comes. Notably, maintaining this state often slows performance on the ongoing task even when the intention cannot yet be fulfilled (Ball et al., 2014; Einstein & McDaniel, 2005; Smith, 2003). Together, these findings converge on the idea that intentions can remain internally active despite the presence of external support, consistent with duplicative strategies of offloading.

Consistent with this idea, Dupre et al. (2024) had participants complete a PM task in which they were periodically probed about their thoughts during a retention interval. Despite being told that an external store would be perfectly reliable, participants in the reminder condition reported just as many PM-related thoughts as those without reminders. Additionally, Landsiedel and Gilbert (2015) observed that while reminders reduced activity in brain regions involved in retrieving the retrospective content of intentions, they did not attenuate activation in regions associated with future-oriented processing. These findings suggest that offloading adds a duplicate source of support rather than replacing internal maintenance of future intentions. It is important to note, however, that participants in the Dupre et al. study experienced only a single block with reminder support, which may have limited their opportunity to update expectations about reminder reliability or to disengage from internal maintenance.

### Current study

The goal of the present study was to test whether prospective memories are offloaded in a substitutive or duplicative fashion as a function of perceived reminder reliability. Traditional PM accounts implicitly assume that once intentions are formed, they are maintained internally until executed, regardless of external support. Under this duplicative view, offloading adds a redundant layer. In contrast, a substitutive view characterizes offloading as a shift in reliance, whereby individuals reduce sustained internal maintenance when reliable external aids are available.

To adjudicate between these views, we tested whether reminder reliability altered (a) the extent to which intentions remained present in conscious thought during the retention interval, and (b) vulnerability to performance decrements when reminders were unexpectedly unavailable. Thought probes served as a real-time proxy for whether the intention remained in conscious awareness during the retention interval between encoding and retrieval. Under duplicative offloading, PM-related thought should remain high regardless of reminder reliability. Under substitutive offloading, reliable reminders should reduce PM-related thought, and PM performance should fall below no-reminder levels when reminders are withdrawn. We did not make a priori predictions about whether freed attention resulting from reduced PM-related thought (if observed) would be redistributed toward increased focus on the ongoing task, increased task-unrelated thought, or a combination of both.

## Methods

### Research transparency statement

All research followed ethical guidelines and was approved by the University of Texas at Arlington Institutional Review Board (IRB). The study included two reminder availability conditions (Present vs. Absent), conducted successively as separate experiments. Each was preregistered independently before data collection and initially reported as a standalone design as part of a Masters’ Thesis. All hypotheses, methods, and analyses were preregistered. Although not preregistered, for clarity and brevity, we added availability as a fixed factor in the model given their methodological similarity. Data and analysis scripts are available via the Open Science Framework (https://osf.io/xj5mk/).

### Study design, participants, and sampling plan

This was a 2 (Session: Trust vs. Critical; *within-subjects*) × 3 (Reminder Reliability: 0%, 50%, 100%; *between-subjects*) × 2 (Reminder Availability: Present vs. Absent; *between-subjects*) mixed-factorial design. Participants were University of Texas at Arlington undergraduates who received course credit. Eligibility required age ≥ 17 years and English fluency. An a priori power analysis targeting a small-to-medium interaction effect for the Session × Reminder Reliability interaction (*f* = 0.192, *α* =.05, 1 – *β* = 0.80) suggested a minimum of 135 participants per availability condition. This interaction was selected a priori as the primary theoretically diagnostic effect of interest. The target sample size was set at 150 participants (50 per reliability condition). In the Reminder-Present condition, 167 participants were recruited and 163 retained after exclusions. In the Reminder-Absent condition, 164 were recruited and 157 retained. The final combined sample comprised 320 participants: 108 in the 0% condition, 109 in the 50% condition, and 103 in the 100% condition.

### Materials

Materials followed Dupre et al. (2024). A total of 758 words/nonwords were selected from the English Lexicon Project (Balota et al., 2007). Fifteen of each were used in practice, and 364 of each appeared in the primary task. Sixteen additional words formed eight PM target word pairs (e.g., DUNE–MANOR), half presented in Session 1, half in Session 2. In reminder conditions, word pairs appeared at the top of the screen in yellow font.

### Procedure

**Fig. 1:**
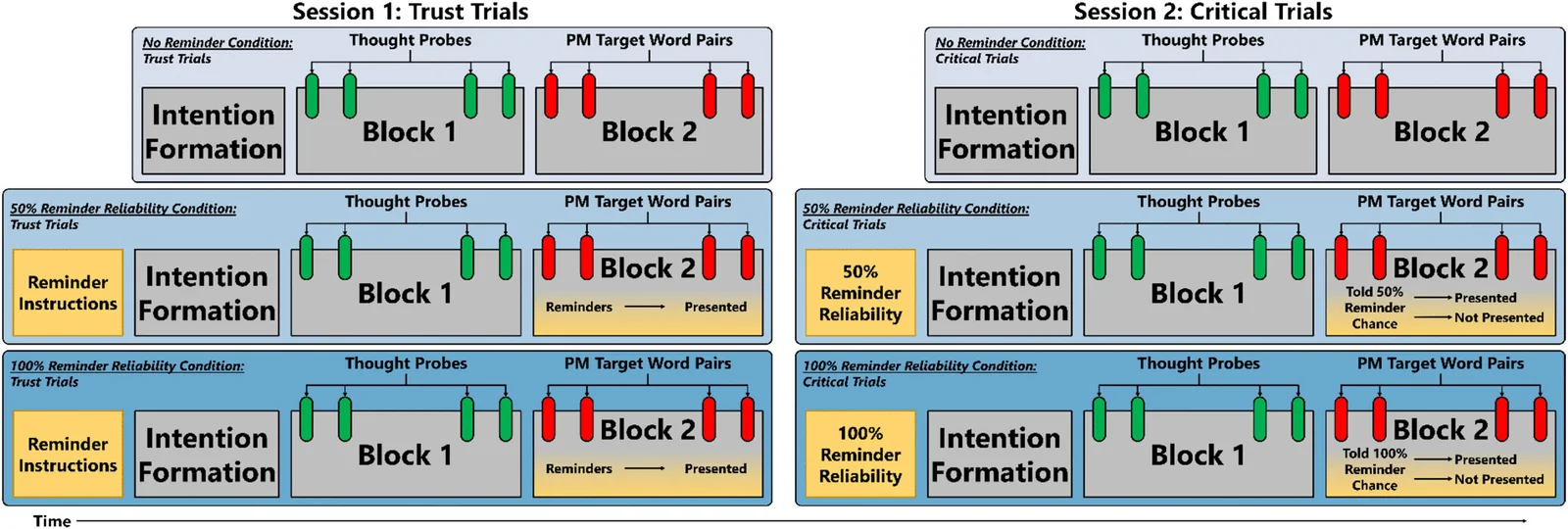
Experimental design overview. Schematic representation of the two-session experimental procedure. Each row represents a Reminder Reliability condition (0%, 50%, or 100%), with Trust trials on the left and Critical trials on the right. During Trust trials (Session 1), participants in the 50% and 100% groups were told that reminders would be available and consistently received them in Block 2. During Critical trials (Session 2), participants in the reminder conditions were told there was a 50% or 100% chance of receiving reminders; however, reminders were only presented in the Reminder-Present condition (bottom row of each Reliability panel). Reminder Availability was manipulated between subjects and is reflected in the yellow boxes beneath Block 2. Thought probes were administered during Block 1 of both sessions, and prospective memory (PM) accuracy was measured in Block 2. Time flows from left to right

Participants completed a two-session PM task embedded within a lexical decision paradigm (see Fig. 1). Each session consisted of an encoding phase, a retention interval with intermittent thought probes (Block 1), and a retrieval phase with embedded PM targets (Block 2). Session 1 served as a *trust trial* phase. Participants in the 50% and 100% reminder reliability conditions were informed that reminders would be available at retrieval and consistently received them during Block 2. Participants in the no-reminder condition received no reminder instructions. Session 2 served as the *critical trial* phase. Before encoding, participants in the reminder conditions were informed that reminders would appear with either 50% or 100% reliability. Reminder availability at retrieval (Present vs. Absent) was manipulated between subjects, such that some participants who expected reminders did not receive them during Block 2. Participants in the no-reminder condition again received no reminder instructions. Thought probes were administered during Block 1 of both sessions, prior to the retrieval phase.

#### Practice and intention instruction

Following practice of the ongoing lexical decision task (word vs. nonword judgments), participants were instructed to study PM word pairs (e.g., DUNE – MANOR). Their intention was to press the spacebar when seeing the target word (e.g., DUNE) during the ongoing task and then type the associated word (e.g., MANOR). PM targets only appeared in Block 2, and the transition was marked by a demographic questionnaire. Participants were told they would receive thought probes during Block 1, asking whether they were: (1) focused on the ongoing task, (2) thinking about the PM task, or (3) off task. A comprehension quiz followed. All questions had to be answered correctly, with incorrect answers triggering a review of instructions.

#### Trust trials (Session 1)

Block 1 included 201 lexical trials and four probes (one every 50 trials), followed by demographic questions. Block 2 included 162 lexical trials and four PM targets (one every 40 trials). Before PM target encoding, participants in the 50% and 100% conditions were validly told that reminders would appear in Block 2 and shown a preview of the reminder format (yellow word pairs above stimuli). Following encoding, they were asked whether they planned to use reminders. Participants in the no-reminder condition were not told about reminders. In Block 2, reminders were always present for the 50% and 100% conditions. PM word pairs were counterbalanced across sessions.

#### Critical trials (Session 2)

Session 2 began with instructions stating that earlier PM targets were no longer relevant. The procedure matched Session 1 but added the reminder reliability and availability manipulations. For reliability, before encoding, participants in the 50% and 100% condition were told there was either a 50% or a 100% chance that reminders would be shown. The no-reminder condition received no reminder instructions. For availability, reminders were shown in Block 2 in the *reminder-present* condition, but not in the *reminder-absent* condition, regardless of expectation.

#### Attention checks

At the end of each session, participants completed an attention check (e.g., “press 3 if you are paying attention”).

#### Post-experimental phase

After Session 2, participants completed a questionnaire assessing memory for instructions and task experience, followed by a recognition test with eight old and eight new PM word pairs.

### Preregistered dependent variables

The primary dependent variables included the proportion of each thought probe response (PM-related, task-related, or task-unrelated) in Block 1 and PM performance during Block 2. PM performance was assessed using two metrics: the proportion of PM targets that elicited any response (PM noticing) and the proportion of targets that were noticed *and* followed by a correctly typed response (PM accuracy). As results were similar, we report only the latter. Secondary measures (see Online Supplemental Materials) included lexical decision speed and accuracy, false alarms (“spacebar”) to non-target trials, and recognition memory.

### Data exclusion

Participants were excluded from analysis if they met any of the following preregistered criteria: failing attention checks (*n* = 0), failing to detect any PM targets *and* failing to remember the PM task instructions on the post-experimental questionnaire (*n* = 1),^1^ achieving less than 60% accuracy on the ongoing task during the PM Block (*n* = 7), or exceeding a 20% false alarm rate on ongoing task trials (*n* = 2). The combined exclusion counts (*n* = 10) yielded a final dataset of 320 included participants.

### Statistical analysis strategy

A series of three-way ANOVAs examined the effects of Session (Trust vs. Critical; within-subjects), Reminder Reliability (No Reminder, 50%, 100%; between-subjects), and Reminder Availability (Present vs. Absent; between-subjects) on thought probe responses and PM accuracy. Tables 1 and 2 present descriptive statistics and omnibus results, respectively. For brevity, only post hoc comparisons following significant highest-order interactions are reported. These include Bonferroni-corrected *t*-tests, effect sizes (Cohen’s *d*), 95% confidence intervals, and Bayes factors. Consistent with conventional interpretations, Bayes factors between 1 and 3 are treated as equivocal and are not taken as confirmatory evidence for either hypothesis (Wagenmakers et al., 2018). Accordingly, theoretical interpretation emphasizes contrasts yielding convergent evidence across effect sizes, confidence intervals, and Bayes factors.

**Table 1:** Means and standard errors for each dependent variable (DV) by session, reliability, and availability

DV	Reliability	Availability	Session	
			Trust	Critical
PM-Related Thoughts	100%	Present	0.26 (.04)	0.16 (.04)
		Absent	0.18 (.04)	0.13 (.04)
	50%	Present	0.21 (.03)	0.30 (.05)
		Absent	0.23 (.04)	0.32 (.05)
	No Reminder	Present	0.28 (.04)	0.45 (.05)
		Absent	0.29 (.05)	0.36 (.05)
Task-Related Thoughts	100%	Present	0.71 (.04)	0.73 (.04)
		Absent	0.78 (.04)	0.78 (.05)
	50%	Present	0.73 (.04)	0.59 (.05)
		Absent	0.68 (.04)	0.64 (.05)
	No Reminder	Present	0.65 (.05)	0.47 (.05)
		Absent	0.65 (.04)	0.54 (.05)
Task-Unrelated Thoughts	100%	Present	0.04 (.01)	0.11 (.03)
		Absent	0.03 (.01)	0.09 (.03)
	50%	Present	0.06 (.02)	0.11 (.03)
		Absent	0.09 (.02)	0.04 (.02)
	No Reminder	Present	0.07 (.02)	0.08 (.03)
		Absent	0.06 (.02)	0.10 (.03)
PM Accuracy	100%	Present	0.61 (.05)	0.76 (.04)
		Absent	0.71 (.04)	0.15 (.04)
	50%	Present	0.69 (.04)	0.75 (.04)
		Absent	0.63 (.04)	0.21 (.04)
	No Reminder	Present	0.31 (.04)	0.24 (.04)
		Absent	0.29 (.05)	0.29 (.04)

Values reflect means and standard errors, reported as *M* (*SE*), for each DV by reliability condition, availability (Present vs. Absent), and session (Trust vs. Critical)

**Table 2:** Omnibus ANOVA results for each dependent variable (DV)

DV	Effect	*η*^2^_G_	90% CI	*F*	*df*_GG_	*df*_res_	*p*
PM-Related Thoughts	Reliability	.043	[.012,.083]	10.84	2	314	<.001
	Availability	.001	[.000,.016]	0.63	1	314	.427
	Session	.005	[.000,.026]	4.58	1	314	.033
	Reliability × Availability	.002	[.000,.014]	0.55	2	314	.576
	Reliability × Session	.017	[.000,.044]	7.95	2	314	<.001
	Availability × Session	.000	[.000,.008]	0.13	1	314	.717
	Reliability × Availability × Session	.002	[.000,.014]	1.05	2	314	.350
Task-Related Thoughts	Reliability	.044	[.012,.083]	10.93	2	314	<.001
	Availability	.002	[.000,.019]	0.97	1	314	.325
	Session	.013	[.000,.041]	11.74	1	314	<.001
	Reliability × Availability	.002	[.000,.010]	0.37	2	314	.690
	Reliability × Session	.009	[.000,.029]	3.99	2	314	.019
	Availability × Session	.001	[.000,.015]	0.94	1	314	.334
	Reliability × Availability × Session	.002	[.000,.011]	0.77	2	314	.463
Task-Unrelated Thoughts	Reliability	.000	[.000,.001]	0.08	2	314	.927
	Availability	.000	[.000,.011]	0.18	1	314	.673
	Session	.008	[.000,.033]	9.90	1	314	.002
	Reliability × Availability	.001	[.000,.007]	0.22	2	314	.806
	Reliability × Session	.007	[.000,.025]	3.90	2	314	.021
	Availability × Session	.002	[.000,.018]	2.01	1	314	.158
	Reliability × Availability × Session	.006	[.000,.025]	3.78	2	314	.024
PM Accuracy	Reliability	.160	[.101,.219]	40.91	2	314	<.001
	Availability	.079	[.038,.131]	37.02	1	314	<.001
	Session	.048	[.017,.093]	59.15	1	314	<.001
	Reliability × Availability	.051	[.016,.092]	11.49	2	314	<.001
	Reliability × Session	.016	[.000,.042]	9.33	2	314	<.001
	Availability × Session	.084	[.042,.137]	106.93	1	314	<.001
	Reliability × Availability × Session	.065	[.025,.110]	40.22	2	314	<.001

Effect sizes are generalized eta squared ${\widehat{\eta }}_{G}^{2}$. *CI* confidence interval; *GG* Greenhouse–Geisser corrected

## Results

### Thought probe responses

**Fig. 2:**
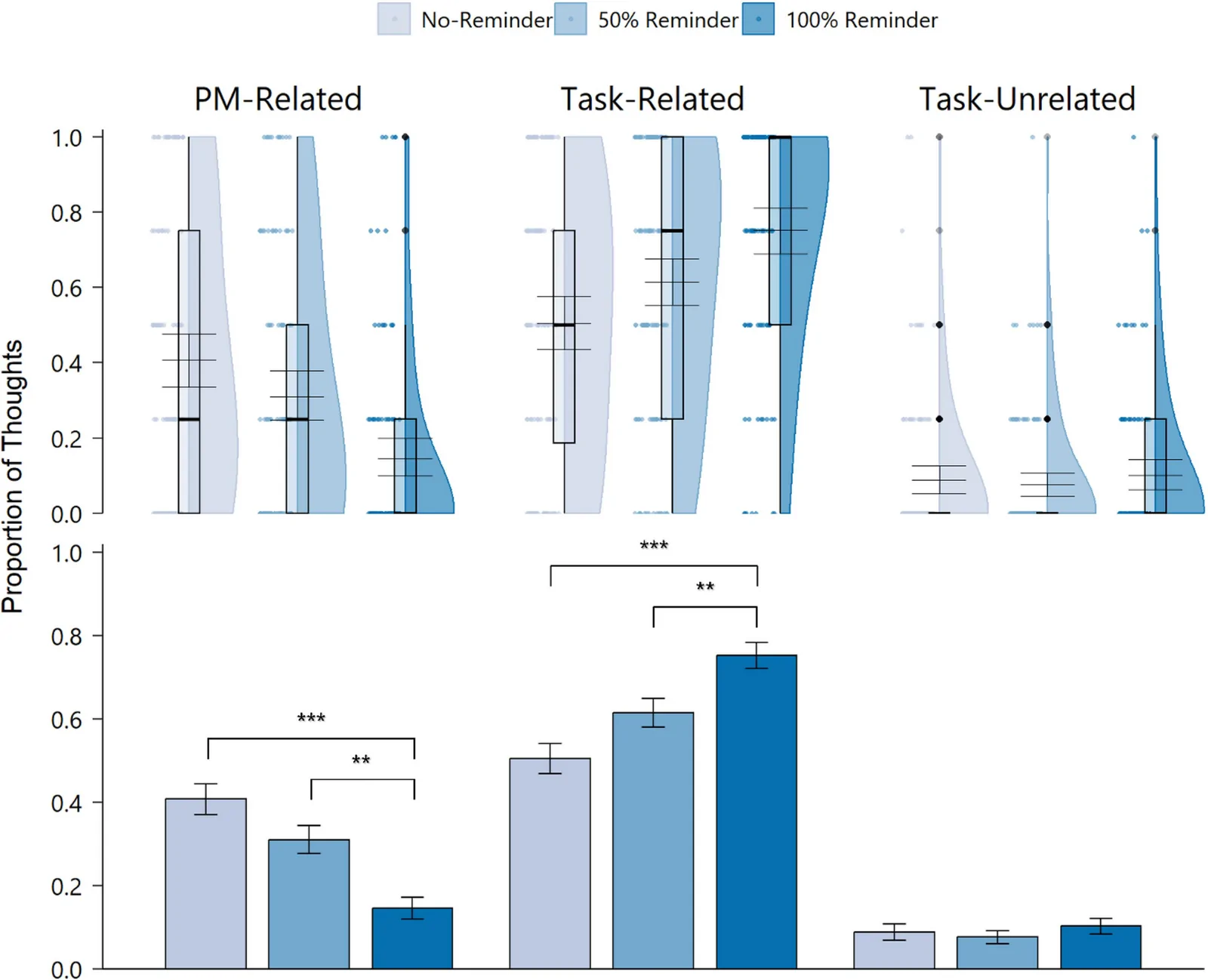
Thought probe responses by reminder condition during intention retention. Raincloud plots (**top**) and corresponding bar plots (**bottom**) showing the proportion of thought probe responses categorized as PM-related, Task-related, or Task-unrelated, by reminder condition. Each raincloud displays individual data points (jittered), a smoothed violin density, a central boxplot, and bootstrapped confidence intervals. Bars reflect group means with ± 1 standard error. Reminder condition is coded by increasing external support: No-Reminder (lightest blue), 50% Reminder (medium blue), and 100% Reminder (darkest blue). Thought probe data were collected during the retention interval in Session 2. *** *p* <.001, ** *p* <.01, * *p* <.05

Figure 2 displays the average thought probe responses for each condition during session 2.

#### Prospective memory (PM)-related thoughts

Main effects of Reliability and Session on PM-related thoughts were qualified by a significant Reliability × Session interaction (Table 2). Post hoc comparisons within each session showed that during trust trials (Session 1), no significant differences were observed between reliability conditions [all *p*s >.20, all BFs_10_ < 1]. During critical trials (Session 2), participants in the 100% condition reported fewer PM-related thoughts than those in the No-Reminder condition [*t*(314) = 5.57, *p* <.001, *d* = 0.63, 95% CI [0.40, 0.85], BF_10_ > 1000], and the 50% condition [*t*(314) = 3.51, *p* =.001, *d* = 0.40, 95% CI [0.17, 0.62], BF_10_ = 110.17]. The contrast between the No-Reminder and 50% conditions yielded a small effect size and equivocal evidence [*t*(314) = 2.09, *p* =.093, *d* = 0.24, 95% CI [0.01, 0.46], BF_10_ = 0.88], indicating that PM-related thought in the 50% condition was numerically intermediate, with insufficient evidence to distinguish it reliably from the No-Reminder condition. The Reliability × Session × Availability interaction was not significant, which was anticipated given that the availability manipulation (i.e., Block 2 of critical trials) was not introduced until after thought probes were recorded (i.e., Block 1 of both sessions). Thus, 100% reliable reminders reduced PM-related thoughts during critical trials.

#### Task-related thoughts

The significant main effects of Reliability and Session on task-related thoughts were qualified by a significant Reliability × Session interaction. Post hoc analyses revealed that no significant differences emerged across reliability conditions during trust trials [Session 1; all *p*s ≥.058, BFs_10_ < 1.53]. During critical trials (Session 2), participants in the 100% condition reported more ongoing-task thoughts than those in the No-Reminder condition [*t*(314) = 5.10, *p* <.001, *d* = 0.58, 95% CI [0.35, 0.80], BF_10_ > 1000], and the 50% condition [*t*(314) = 2.84, *p* =.013, *d* = 0.32, 95% CI [0.10, 0.54], BF_10_ = 8.43]. The contrast between the 50% and No-Reminder conditions yielded a small effect size and equivocal evidence of a difference between the two [*t*(314) = 2.30, *p* =.057, *d* = 0.26, 95% CI [0.04, 0.48], BF_10_ = 1.45]. The Reliability × Session × Availability interaction was not significant. Thus, 100% reliable reminders increased task-related thoughts during critical trials.

#### Task-unrelated thoughts

The significant main effects of Reliability and Session on task-unrelated thoughts were qualified by a significant Reliability × Session interaction. Post hoc analyses conducted separately for each session revealed that no significant differences emerged across reliability conditions during trust trials [Session 1; all *p*s ≥.12, all BFs_10_ < 1]. Likewise, during critical trials (Session 2), no pairwise comparisons reached significance [all *p*s >.50, all BFs_10_ < 1], again supporting the null. A three-way Reliability × Availability × Session interaction was also significant. However, follow-up contrasts conducted separately by availability condition revealed no statistically significant pairwise differences [all *p*s ≥.10, BFs_10_ < 2.20]. Across analyses, Bayes factors consistently favored neither hypothesis, supporting the conclusion that reminder reliability did not meaningfully alter task-unrelated thought.

#### PM accuracy

**Fig. 3:**
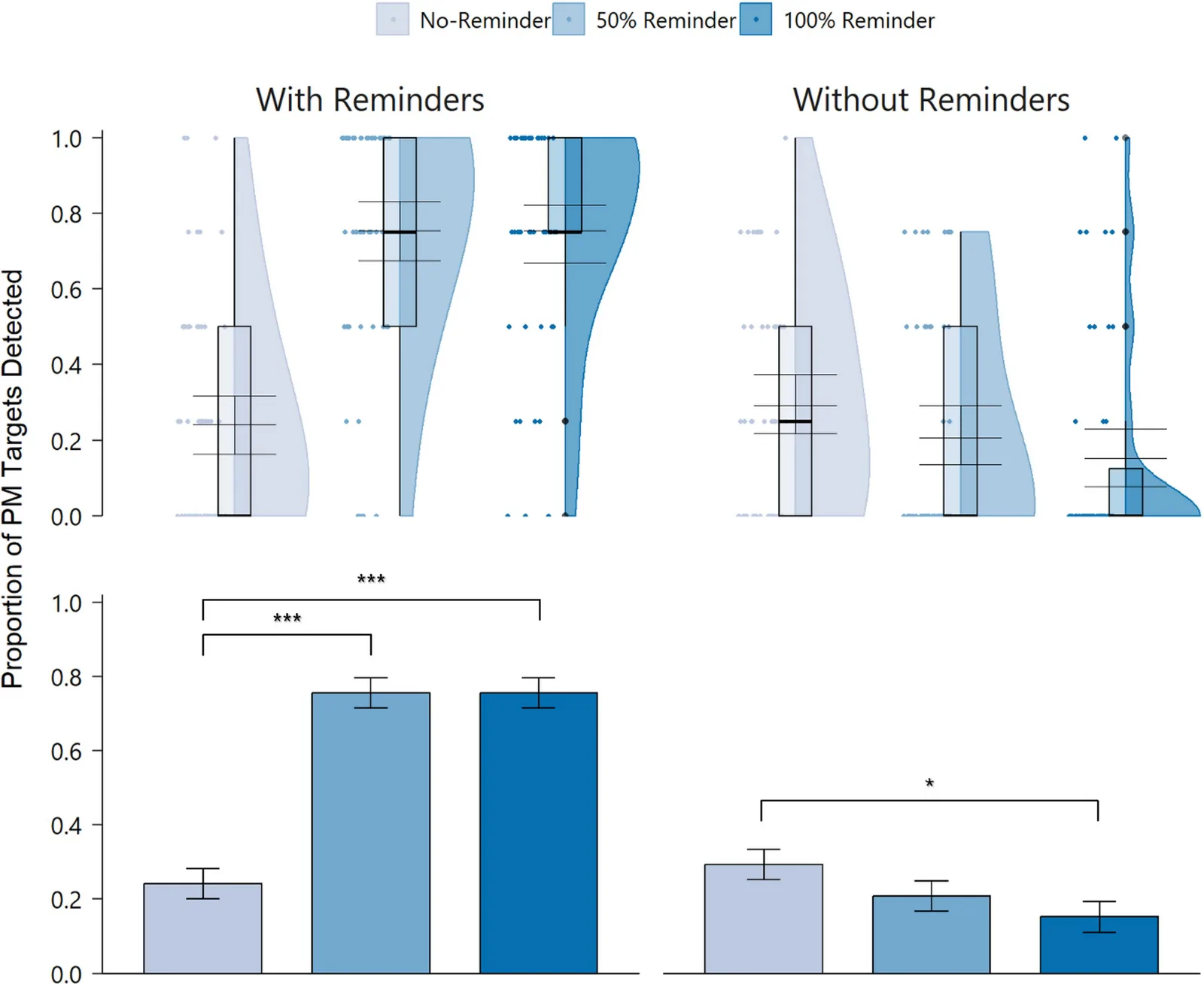
Prospective memory (PM) accuracy by reminder reliability and availability condition. The figure displays PM accuracy performance across reminder reliability conditions (No-Reminder, 50% Reminder, 100% Reminder), separately for trials where external reminders were available (**left**) and unavailable (**right**). Each raincloud displays individual data points (jittered), a smoothed violin density, a central boxplot, and bootstrapped confidence intervals. Bars reflect group means with ± 1 standard error. *** *p* <.001, ** *p* <.01, * *p* <.05

Figure 3 displays PM accuracy by Reliability and Availability. All main effects and interactions were significant, including the Reliability × Availability × Session interaction. Post hoc analyses were conducted separately by Session and Availability.

Recall that reminders were presented for the 50% and 100% condition in both the availability conditions during trust trials (Session 1). Results showed that in Session 1, both the 50% and 100% reliability conditions outperformed the No-Reminder condition when reminders were later available in Session 2 [50% vs. No-Reminder: *t*(314) = 6.37, *p* <.001, *d* = 0.72, 95% CI [0.49, 0.95], BF_10_ > 1000; 100% vs. No-Reminder: *t*(314) = 4.94, *p* <.001, *d* = 0.56, 95% CI [0.33, 0.78], BF_10_ > 1000]. There was no difference between reminder conditions when reminders were later available [50% vs. 100%: *t*(314) = 1.32, *p* =.386, *d* = 0.15, 95% CI [–0.07, 0.37], BF_10_ = 0.43]. The same pattern was observed when reminders were later unavailable in Session 2, with both reminder conditions outperforming the No-Reminder condition (all *p*s <.001, all BFs_10_ > 1000) and no difference between reminder conditions.

The pattern diverged during critical trials (Session 2), as expected, given that reminders were presented for the 50% and the 100% condition in the available condition, but not the unavailable condition. Results showed that when reminders were available, both the 50% and the 100% reliability conditions again outperformed the No-Reminder condition [50% vs. No-Reminder: *t*(314) = 9.07, *p* <.001, *d* = 1.02, 95% CI [0.79, 1.26], BF_10_ > 1000; 100% vs. No-Reminder: *t*(314) = 8.91, *p* <.001, *d* = 1.01, 95% CI [0.77, 1.24], BF_10_ > 1000], and there again was no difference between reminder conditions [50% vs. 100%: *t*(314) < 0.01, *p* = 1.00, *d* < 0.01, 95% CI [–0.22, 0.22], BF_10_ = 0.20].

However, when reminders were unavailable, performance in the 100% condition, but not the 50% condition, was significantly lower than in the No-Reminder condition [50% vs. No-Reminder: *t*(314) = 1.47, *p* =.309, *d* = 0.17, 95% CI [–0.06, 0.39], BF_10_ = 0.54; 100% vs. No-Reminder: *t*(314) = 2.40, *p* =.045, *d* = 0.27, 95% CI [0.05, 0.49], BF_10_ = 2.78]. There was no difference between reminder conditions [50% vs. 100%: *t*(314) = 0.95, *p* =.609, *d* = 0.11, 95% CI [–0.11, 0.33], BF_10_ = 0.31]. Thus, 100% reliable reminders reduced PM accuracy relative to no reminders when they were unexpectedly removed. However, the corresponding Bayes factor (BF_10_ = 2.78) indicates equivocal evidence, and this effect should be interpreted cautiously as suggestive rather than definitive.

## General discussion

It has been suggested that retrospective memory evolved, in part, to serve future-oriented behavior (Nairne & Pandeirada, 2008). Indeed, research shows that retrospective and prospective thought recruit overlapping neural substrates (Addis et al., 2007), and off-task thought is frequently oriented toward the future rather than the past (e.g., Baird et al., 2011; Smallwood et al., 2009). This prioritization and above-baseline future-oriented focus may be adaptive, insofar as it increases the likelihood of triggering ecphory from cues in the environment (Goschke & Kuhl, 1993; Marsh et al., 1998; Martin et al., 2011; Underwood et al., 2015). Duplicating intentions across internal and external sources is therefore sensible, especially when external reliability is uncertain (e.g., a phone low on battery). However, humans are also cognitive misers (Kool et al., 2010), and redundantly encoding, maintaining, and/or retrieving intentions can be metabolically inefficient. Substitutive offloading would consequently be the most optimal decision in terms of balancing memory and effort. The present study was designed to tease apart whether individuals adopt duplicative strategies that maintain internal memory alongside external reminders, or substitutive strategies that rely on the external store alone.

Consistent with Dupre et al. (2024), PM-related thoughts during trust trials did not differ across conditions, despite both reminder conditions outperforming the 0% condition at retrieval. This suggests an initial reliance on duplicative offloading. However, after gaining experience with reliable external support, the strategies diverged. In the 50% condition, participants maintained high levels of PM-related thought on critical trials, with no reduction in future-oriented thinking compared to the 0% condition. Although PM performance declined when reminders were withdrawn, it did not fall below no-reminder levels. Together, these findings suggest that uncertainty limits disengagement from internal intention maintenance, such that individuals continue to actively think about intentions even when external support is expected. As a result, the 50% condition often fell between full reliance on reminders and no external support. This pattern is consistent with a more duplicative strategy than that observed with fully reliable reminders, although the intermediate position of the 50% condition warrants cautious interpretation given equivocal evidence in several pairwise contrasts.

In contrast, participants in the 100% condition appear to have updated their beliefs (Dunlosky & Hertzog, 2000) about the efficacy of reminders and the necessity to effortfully encode and maintain them, showing two distinct patterns consistent with greater substitutive use of reminders. First, PM-related thoughts decreased, and task-related thoughts increased relative to the no-reminder condition, reflecting a redistribution of reported thoughts during the retention interval. Such a reduction in reported PM-related thought challenges classical Zeigarnik-style views of persistent goal activation (Zeigarnik, 1927) and instead suggests that intention maintenance is dynamically tempered by expectations of environmental support. Second, PM accuracy dropped significantly below the no-reminder condition when reminders were unexpectedly withdrawn. However, the Bayes factor for this contrast was modest (BF₁₀ = 2.78), and this effect should be interpreted alongside the stronger evidence from the thought probe redistribution. If internal representations had been maintained in parallel with external reminders, performance should have at least matched no-reminder levels, as was observed in the 50% condition. Together, these findings indicate that fully reliable reminders promoted a shift toward substitutive offloading, characterized by reduced internal maintenance of intentions during the retention interval and a corresponding redistribution of cognitive resources. This shift, in turn, left performance vulnerable when external support was unavailable.

### Possible mechanisms driving offloading effects

One contributor to the observed vulnerability following the unexpected withdrawal of reliable reminders may be reduced encoding or maintenance effort. Expectations of external aid can lower study investment and impair unaided performance in PM (Peper & Ball, 2025). More broadly, work on recall and recognition shows that people update their beliefs as they acquire metacognitive knowledge about prior performance and likely memory success (Dunlosky & Hertzog, 2000; Koriat & Goldsmith, 1996). From an offloading perspective, such metacognitive adjustments may instantiate an effort-minimization strategy, whereby internal encoding is downregulated once external support is judged sufficient (Kelly & Risko, 2022; Sachdeva & Gilbert, 2020). At the same time, the performance cost following reminder withdrawal is not uniquely diagnostic of substitution and may also reflect retrieval-level factors, such as reminder-contingent retrieval strategies or sensitivity to violated expectations (Kelly & Risko, 2022; Peper & Ball, 2025).

Greater reliance on external storage at the expense of sustained internal maintenance is also consistent with broader theoretical accounts emphasizing distributed cognition and transactive memory systems, in which cognitive processes extend beyond the individual and are shaped by interactions with external tools and social partners (Hollan et al., 2000; Kirsh, 2010). Within this framework, memory is scaffolded by trusted external sources, such that individuals encode both where that information resides and who is responsible for remembering it (Wegner, 1987; Wegner et al., 1985). This division of cognitive labor allows storage and retrieval demands to be offloaded to reliable external stores. Analogous patterns appear in technological contexts, including GPS systems (Gardony et al., 2015; Ishikawa et al., 2008), note-taking sources (Burnett & Richmond, 2025; Kelly & Risko, 2022), and digital search tools. For example, when individuals expect information to be accessible online, they show reduced memory for content but enhanced memory for its location (Sparrow et al., 2011).

Lastly, some have likened offloading to directed forgetting, where writing something down leads to reduced processing of that information (Eskritt & Ma, 2014). However, recent evidence suggests that offloaded content may not be fully suppressed or forgotten. Gist-level information often remains accessible (Lu et al., 2020; but see Magen & Tomer-Offen, 2025), and neuroimaging data show residual activation during encoding (Runge et al., 2021; Tsai, 2023). We speculate that in both retrospective and prospective memory paradigms, people may encode a reformulated task goal that retains the knowledge that the information exists and where it can be found. Rather than actively maintaining this content in working memory, the reminder system itself may function as the retrieval cue for the underlying goal (Peper et al., 2023). In this sense, the new goal may persist in an activity-silent state over the retention interval, analogous to models of latent working memory representations that can be reactivated when needed (Rose et al., 2016). This interpretation offers one possible account that helps to explain how offloaded intentions may be absent from moment-to-moment thought but remain accessible to guide behavior when the appropriate cue is encountered.

### Limitations and broader implications

A key strength of this study was the use of real-time thought probes to assess momentary PM-related thought, providing a more direct and temporally precise measure of cognitive state than retrospective reports or behavioral markers alone. Though probe reactivity is a concern, they can reliably capture internal thought without disrupting performance (Kane et al., 2021; Wiemers & Redick, 2019), and they converge with implicit measures like lure-related reaction times (Dupre et al., 2024). However, future work may consider examining study duration (Peper & Ball, 2025) or using probes during encoding (Scullin et al., 2018) and/or retrieval (Rummel et al., 2022) to better track when representational shifts occur. It is also important to note that probe response categories are mutually exclusive, such that a shift from one category must necessarily be redistributed elsewhere. Accordingly, effects on individual thought categories should be interpreted as shifts in the distribution of conscious thought rather than changes in independent processes.

Finally, the brief retention interval and experimenter-imposed reminders used here limit ecological validity. Real-world intentions often unfold over longer timeframes, are embedded in shifting goals, and rely on self-initiated processes (Gilbert, 2015; Scott & Gilbert, 2024). Investigating how internal and external strategies interact under these more naturalistic conditions may improve ecological validity and offer a powerful lens into the flexibility of internal memory representations. Understanding when and why people shift between internal and external systems may yield practical insights for high-stakes environments like aviation or healthcare (Loft et al., 2013; Parasuraman & Riley, 1997), where effective supports must balance reliability with redundancy to ensure offloading enhances rather than undermines performance.

## Supplementary Information

Below is the link to the electronic supplementary material.

Supplementary file1 (PDF 143 kb)

## Data Availability

The data and experimental materials are available via the Open Science Framework (https://osf.io/xj5mk/).

## Footnotes

1 In the post-experimental questionnaire, 49 participants received invalid response options for the PM verification question. Forty-six detected at least one target; the remaining three were retained due to unverifiable responses.

## Code availability

All analysis codes are available via the Open Science Framework (https://osf.io/xj5mk/).
