# Supplementary material for: Let it go: How trusted reminders alter intention maintenance
Source: Psychon Bull Rev. 2026 Aug 18;33(7):211. doi: 10.3758/s13423-026-02985-6 (PMC13486017; doi:10.3758/s13423-026-02985-6)
Supplement: Supplementary file 1 [file 13423_2026_2985_MOESM1_ESM.pdf]

Supplemental Results

Data Transformations

For analyses of ongoing task performance, the first three trials and the three trials following each thought probe and PM target were excluded. Reaction times exceeding 3.0 standard deviations from each participant's mean were trimmed.

Table 1: Means and Standard Errors of PM Noticing, PM False Alarms, OGT Accuracy, and OGT RT, by Reliability, Availability, Session, and Block.

**DV**	**Reliability**	**Availability**	**Session**			
			Trust		Critical	
			Block 1	Block 2	Block 1	Block 2
PM Noticing	100%	Present		0.635 (.047)		0.764 (.041)
		Absent		0.725 (.041)		0.206 (.046)
	50%	Present		0.705 (.043)		0.777 (.040)
		Absent		0.660 (.042)		0.335 (.049)
	No Reminder	Present		0.473 (.047)		0.359 (.051)
		Absent		0.396 (.048)		0.462 (.049)
PM False Alarms	100%	Present	0.077 (.037)	0.481 (.462)	0.019 (.019)	0.019 (.019)
		Absent	0.020 (.020)	0.196 (.177)	0.078 (.038)	0.078 (.047)
	50%	Present	0.125 (.092)	0.571 (.571)	0.107 (.061)	0.018 (.018)
		Absent	0.132 (.061)	0.491 (.454)	0.076 (.046)	0.264 (.077)
	No Reminder	Present	0.036 (.026)	0.091 (.039)	0.055 (.031)	0.145 (.048)
		Absent	0.038 (.026)	0.189 (.066)	0.038 (.026)	0.170 (.075)
OGT Accuracy	100%	Present	0.867 (.010)	0.869 (.011)	0.877 (.010)	0.877 (.010)
		Absent	0.894 (.009)	0.883 (.010)	0.890 (.009)	0.885 (.009)
	50%	Present	0.887 (.007)	0.880 (.008)	0.883 (.008)	0.885 (.008)
		Absent	0.883 (.010)	0.881 (.010)	0.884 (.009)	0.887 (.009)
	No Reminder	Present	0.866 (.009)	0.877 (.009)	0.870 (.009)	0.870 (.009)
		Absent	0.880 (.008)	0.872 (.009)	0.873 (.009)	0.875 (.009)
OGT RT (ms)	100%	Present	1110 (47.7)	1160 (49.6)	980 (35.5)	1096 (47.4)
		Absent	1056 (35.0)	1119 (38.2)	974 (36.7)	986 (43.9)
	50%	Present	1056 (37.0)	1085 (36.3)	942 (34.2)	1022 (32.7)
		Absent	980 (39.3)	1028 (34.7)	881 (25.7)	898 (26.4)
	No Reminder	Present	1158 (58.6)	1110 (40.1)	989 (35.4)	1022 (35.9)
		Absent	1070 (57.2)	1062 (44.5)	908 (35.3)	993 (35.5)

Note. Values reflect means and standard errors, reported as $M(SE)$, for each DV by reliability condition, availability (Present vs. Absent), session (Trust vs. Critical), and block (1 vs. 2). PM Notice is collapsed across blocks.

Table 2: Recognition Hits (Block 2) and False Alarms by Reliability and Availability.

**Reliability**	**Availability**	**Session**		**False Alarms**
		**Recognition Hits**		
		**Trust**	**Critical**	
No Reminder	Present	0.764 (.120)	0.850 (.099)	0.098 (.125)
	Absent	0.708 (.156)	0.821 (.130)	0.068 (.086)
50%	Present	0.830 (.117)	0.862 (.114)	0.038 (.059)
	Absent	0.835 (.150)	0.854 (.116)	0.080 (.131)
100%	Present	0.880 (.097)	0.885 (.104)	0.029 (.063)
	Absent	0.848 (.146)	0.794 (.150)	0.096 (.140)

Note. Recognition Hits reflect Block 2 performance during Trust and Critical sessions. False Alarms are collapsed across sessions and blocks. Values are reported as $M(SE)$.

Table 3: Omnibus ANOVA results for all dependent variables.

DV	Effect	$\hat{\eta}^2_G$	90% CI	F	df_{GG}	$df^{\text{GG}}_{\text{res}}$	p
PM Notice	Reliability	.064	[.024, .109]	14.48	2	314	< .001
	Availability	.052	[.019, .097]	23.44	1	314	< .001
	Session	.030	[.007, .067]	36.70	1	314	< .001
	Reliability × Availability	.032	[.005, .067]	6.98	2	314	.001
	Reliability × Session	.011	[.000, .034]	6.79	2	314	.001
	Availability × Session	.046	[.015, .089]	57.65	1	314	< .001
	Reliability × Availability × Session	.065	[.025, .110]	41.44	2	314	< .001
OGT Accuracy	Reliability	.005	[.000, .021]	0.93	2	314	.397
	Availability	.003	[.000, .020]	1.00	1	314	.317
	Session	.000	[.000, .008]	0.78	1	314	.378
	Block	.000	[.000, .005]	0.36	1	314	.550
	Reliability × Availability	.002	[.000, .014]	0.47	2	314	.626
	Reliability × Session	.000	[.000, .001]	0.90	2	314	.406
	Availability × Session	.000	[.000, .006]	0.39	1	314	.534
	Reliability × Block	.000	[.000, .000]	0.60	2	314	.547
	Availability × Block	.000	[.000, .011]	1.91	1	314	.168
	Session × Block	.000	[.000, .007]	0.97	1	314	.326
	Reliability × Availability × Session	.000	[.000, .001]	0.92	2	314	.399
	Reliability × Availability × Block	.000	[.000, .002]	1.24	2	314	.290
	Reliability × Session × Block	.000	[.000, .000]	0.59	2	314	.555
	Availability × Session × Block	.000	[.000, .009]	1.68	1	314	.195
	Reliability × Availability × Session × Block	.000	[.000, .001]	1.67	2	314	.190
OGT RT	Reliability	.011	[.000, .034]	2.31	2	314	.101
	Availability	.012	[.000, .040]	5.04	1	314	.026
	Session	.034	[.009, .073]	100.95	1	314	< .001
	Block	.005	[.000, .025]	20.54	1	314	< .001
	Reliability × Availability	.000	[.000, .001]	0.07	2	314	.930
	Reliability × Session	.000	[.000, .000]	0.39	2	314	.680
	Availability × Session	.000	[.000, .004]	0.12	1	314	.729
	Reliability × Block	.001	[.000, .007]	2.18	2	314	.115
	Availability × Block	.000	[.000, .004]	0.16	1	314	.685
	Session × Block	.001	[.000, .014]	7.20	1	314	.008
	Reliability × Availability × Session	.000	[.000, .000]	0.29	2	314	.752
	Reliability × Availability × Block	.001	[.000, .008]	2.46	2	314	.087
	Reliability × Session × Block	.001	[.000, .007]	4.08	2	314	.018
	Availability × Session × Block	.001	[.000, .014]	5.87	1	314	.016
	Reliability × Availability × Session × Block	.001	[.000, .003]	2.21	2	314	.112
Recognition Hits	Reliability	.017	[.000, .044]	3.93	2	314	.021
	Availability	.006	[.000, .028]	2.73	1	314	.100
	Session	.005	[.000, .027]	5.33	1	314	.022
	Reliability × Availability	.003	[.000, .016]	0.68	2	314	.506
	Reliability × Session	.012	[.000, .036]	6.18	2	314	.002
	Availability × Session	.000	[.000, .010]	0.26	1	314	.611
	Reliability × Availability × Session	.001	[.000, .010]	0.72	2	314	.487
Recognition False Alarms	Reliability	.006	[.000, .024]	0.98	2	314	.376
	Availability	.010	[.000, .035]	3.02	1	314	.083
	Reliability × Availability	.022	[.001, .052]	3.55	2	314	.030
PM False Alarms	Reliability	.002	[.000, .011]	1.06	2	314	.346
	Availability	.000	[.000, .000]	0.00	1	314	.979
	Session	.002	[.000, .018]	2.27	1	314	.133
	Block	.003	[.000, .022]	4.33	1	314	.038
	Reliability × Availability	.000	[.000, .000]	0.14	2	314	.867
	Reliability × Session	.001	[.000, .009]	0.79	2	314	.456
	Availability × Session	.000	[.000, .011]	0.52	1	314	.473
	Reliability × Block	.000	[.000, .000]	0.21	2	314	.808
	Availability × Block	.000	[.000, .000]	0.01	1	314	.913
	Session × Block	.002	[.000, .017]	1.88	1	314	.171
	Reliability × Availability × Session	.000	[.000, .002]	0.28	2	314	.752
	Reliability × Availability × Block	.000	[.000, .000]	0.18	2	314	.835
	Reliability × Session × Block	.001	[.000, .006]	0.53	2	314	.589
	Availability × Session × Block	.000	[.000, .010]	0.34	1	314	.561
	Reliability × Availability × Session × Block	.000	[.000, .000]	0.16	2	314	.849

Note. Effect sizes are generalized eta squared ($\hat{\eta}^2_G$). CI = confidence interval. DV = dependent variable. GG = Greenhouse–Geisser corrected.

Table 4: Three-Way Contrasts for PM Noticing: Reliability × Session × Reminder Availability

Session	Availability	Contrast	M_{diff}	SE	t	p	d	95% CI	BF_{10}
Trust	Present	No vs. 50%	–0.23	0.06	–3.74	< .001	–0.42	[–0.65, –0.20]	65.8
		No vs. 100%	–0.16	0.06	–2.56	.030	–0.29	[–0.51, –0.07]	2.72
		50% vs. 100%	0.07	0.06	1.12	.501	0.13	[–0.09, 0.35]	0.35
	Absent	No vs. 50%	–0.26	0.06	–4.16	< .001	–0.47	[–0.69, –0.24]	304
		No vs. 100%	–0.33	0.06	–5.13	< .001	–0.58	[–0.80, –0.35]	> 1000
		50% vs. 100%	–0.07	0.06	–1.01	.568	–0.11	[–0.34, 0.11]	0.36
Critical	Present	No vs. 50%	–0.42	0.06	–6.52	< .001	–0.74	[–0.96, –0.51]	> 1000
		No vs. 100%	–0.41	0.07	–6.21	< .001	–0.70	[–0.93, –0.47]	> 1000
		50% vs. 100%	0.01	0.07	0.19	.980	0.02	[–0.20, 0.24]	0.21
	Absent	No vs. 50%	0.13	0.07	1.94	.128	0.22	[–0.00, 0.44]	0.91
		No vs. 100%	0.26	0.07	3.87	< .001	0.44	[0.21, 0.66]	96
		50% vs. 100%	0.13	0.07	1.95	.127	0.22	[–0.00, 0.44]	1.04

Note. Reported values include mean differences (M_{diff}), standard errors (SE), t-statistics, p-values, Cohen's d, 95% confidence intervals (CI), and Bayes factors (BF_{10}).

PM Noticing

All effects were significant for PM noticing, including the Reliability × Availability × Session interaction. Post hoc analyses were conducted for each session, separately for each availability condition.

Recall that reminders were presented for the 50% and 100% conditions in both availability conditions during trust trials (Session 1). Results showed that in Session 1, both the 50% and 100% reliability conditions yielded higher PM noticing than the No-Reminder condition when reminders were later available in Session 2. There was no difference between reminder conditions when reminders were later available.

During Session 1, when reminders were later unavailable in Session 2, both the 50% and 100% conditions again yielded higher PM noticing than the No-Reminder condition. There was no difference between reminder conditions when reminders were later unavailable.

The pattern diverged during critical trials (Session 2), as expected given that reminders were presented for the 50% and 100% conditions in the available condition, but not the unavailable condition. Results showed that when reminders were available, both the 50% and 100% reliability conditions again yielded higher PM noticing than the No-Reminder condition, and there again was no difference between reminder conditions.

However, when reminders were unavailable, PM noticing in the 100% condition, but not the 50% condition, was significantly lower than in the No-Reminder condition. There was no difference between reminder conditions. Thus, fully reliable reminders reduced PM noticing relative to no reminders when they were unexpectedly removed. However, the corresponding Bayes factors indicate equivocal evidence, and this effect should be interpreted cautiously as suggestive rather than definitive.

Ongoing Task Accuracy

No significant main effects or interactions involving Reliability, Availability, Session, or Block were observed for ongoing task accuracy (Table S3). Descriptive statistics for ongoing task accuracy by Reliability, Availability, Session, and Block are reported in Table S5. Thus, reminder reliability and availability did not reliably affect ongoing task accuracy.

Table 5: Two-Way Contrasts for Ongoing-Task Reaction Time: Session × Block

**Session**	**Contrast**	$\boldsymbol{M}_{\textbf{diff}}$	**SE**	t	p	d	**95% CI**	$\textbf{BF}_{10}$
Trust	Block 1 vs. Block 2	–22.50	11.90	–1.88	.061	–0.21	[–0.43, 0.01]	0.13
Critical	Block 1 vs. Block 2	–57.20	9.78	–5.84	< .001	–0.66	[–0.89, –0.43]	3.50

Note. Reported values include mean differences (M_{diff}), standard errors (SE), t-statistics, p-values, Cohen's d, 95% confidence intervals (CI), and Bayes factors (BF_{10}).

Ongoing-Task Reaction Time

The significant main effects of Session and Block on ongoing-task reaction time were qualified by a significant Session × Block interaction. Post hoc analyses were conducted separately within each session. During Trust trials (Session 1), reaction time did not differ between Block 1 and Block 2, indicating stable ongoing-task performance across blocks. The corresponding Bayes factor favored the null hypothesis.

The pattern diverged during Critical trials (Session 2). Reaction time was reliably faster in Block 2 than in Block 1, indicating pronounced speeding across blocks during the critical session. The associated Bayes factor provided strong evidence for this effect. Thus, ongoing-task reaction time remained stable across blocks during Trust trials but diverged during Critical trials, with faster responses in Block 2 relative to Block 1.

Table 6: Two-Way Contrasts for Recognition Hits: Reliability × Session

**Session**	**Contrast**	$\boldsymbol{M}_{\textbf{diff}}$	**SE**	t	p	d	**95% CI**	$\textbf{BF}_{10}$
Trust	No vs. 50%	–0.39	0.13	–2.96	.009	–0.33	[–0.56, –0.11]	6.22
	No vs. 100%	–0.51	0.13	–3.85	< .001	–0.44	[–0.66, –0.21]	151
	50% vs. 100%	–0.13	0.13	–0.94	.614	–0.11	[–0.33, 0.12]	0.24
Critical	No vs. 50%	–0.09	0.12	–0.75	.732	–0.09	[–0.31, 0.14]	0.20
	No vs. 100%	–0.02	0.12	–0.13	.990	–0.02	[–0.24, 0.21]	0.15
	50% vs. 100%	0.07	0.12	0.61	.814	0.07	[–0.15, 0.29]	0.18

Note. Reported values include mean differences (M_{diff}), standard errors (SE), t-statistics, p-values, Cohen's d, 95% confidence intervals (CI), and Bayes factors (BF_{10}).

Recognition Memory Hits

The significant main effects of Reliability and Session on recognition hits were qualified by a significant Reliability × Session interaction. Post hoc analyses were therefore conducted separately within each session.

During Trust trials (Session 1), recognition hits were reliably higher in both the 50% and 100% reliability conditions compared to the No-Reminder condition, whereas recognition performance did not differ between the two reminder reliability conditions. The associated Bayes factors provided moderate to strong evidence for improved recognition performance in both reminder conditions relative to No Reminder, and favored the null hypothesis for the comparison between the 50% and 100% reliability conditions.

This pattern did not persist during Critical trials (Session 2). Recognition hits did not differ between the No-Reminder condition and either reminder reliability condition, and there was again no difference between the 50% and 100% reliability conditions. The corresponding Bayes factors consistently favored the null hypothesis, indicating evidence for equivalent recognition performance across reliability conditions during critical trials. Thus, the effect of reminder reliability on recognition memory was confined to Trust trials and was absent during Critical trials.

Table 7: Two-Way Contrasts for Recognition False Alarms: Reliability × Reminder Availability

Availability	Contrast	M_{diff}	SE	t	p	d	95% CI	BF_{10}
Present	No vs. 50%	0.478	0.207	2.31	.057	0.26	[0.04, 0.48]	39.6
	No vs. 100%	0.551	0.211	2.61	.026	0.29	[0.07, 0.52]	146
	50% vs. 100%	0.073	0.210	0.35	.936	0.04	[–0.18, 0.26]	0.21
Absent	No vs. 50%	–0.094	0.212	–0.44	.897	–0.05	[–0.27, 0.17]	0.18
	No vs. 100%	–0.218	0.214	–1.01	.568	–0.12	[–0.34, 0.11]	0.35
	50% vs. 100%	–0.123	0.214	–0.58	.834	–0.06	[–0.29, 0.16]	0.18

Note. Reported values include mean differences (M_{diff}), standard errors (SE), t-statistics, p-values, Cohen's d, 95% confidence intervals (CI), and Bayes factors (BF_{10}).

Recognition False Alarms

A significant Reliability × Reminder Availability interaction was observed for recognition false alarm rates. Post hoc analyses were therefore conducted separately within each availability condition.

When reminders were present, false alarm rates were higher in both the 50% and 100% reliability conditions relative to the No-Reminder condition. There was no difference between the two reminder reliability conditions. The corresponding Bayes factors provided strong evidence for differences between the No-Reminder condition and both reminder conditions, while favoring the null hypothesis for the comparison between the 50% and 100% reliability conditions.

When reminders were absent, false alarm rates did not differ across reliability conditions. Bayes factors consistently favored the null hypothesis across all contrasts. Thus, the effect of reminder reliability on recognition false alarms was present only when reminders were available and was absent when reminders were unavailable.

PM False Alarms

For PM false alarms, there was a significant main effect of Block, reflecting higher false alarm rates in Block 2 than in Block 1. No other main effects or interactions were significant. Because no higher-order interactions reached significance, no post hoc contrasts were conducted.

Post-Encoding Reminder Use Intention

We examined how many participants indicated during the post-encoding question that they did not intend to use reminders. This was assessed separately during the trust and critical trials. In the 50% reliability condition, 14% of participants ($n = 15$) reported no intention to use reminders during trust trials, and 15% ($n = 16$) during critical trials. In the 100% reliability condition, 17% of participants ($n = 17$) reported this during trust trials, and 10% ($n = 10$) during critical trials.

To assess whether intention to use reminders differed by reliability condition and session, we conducted a binary logistic regression predicting post-encoding reminder intention (1 = "yes," 0 = "no") from Reliability Condition (50% vs. 100%), Session (Trust vs. Critical), and their interaction. The model included main effects of Condition (coded as 0 = 50%, 1 = 100%), Session (0 = Trust, 1 = Critical), and the Condition × Session interaction.

The overall model did not reveal any significant predictors. The intercept was significant, indicating a general tendency to endorse reminder use, $\beta = 1.84$, SE = 0.28, $z = 6.60$, $p < .001$. There was no significant effect of Condition, $\beta = -0.21$, SE = 0.38, $z = -0.56$, $p = .578$, and no significant effect of Session, $\beta = -0.08$, SE = 0.39, $z = -0.19$, $p = .846$. The Condition × Session interaction was also not significant, $\beta = 0.68$, SE = 0.58, $z = 1.19$, $p = .235$.

Model fit indices were as follows: null deviance = 338.43 on 423 degrees of freedom; residual deviance = 336.18 on 420 degrees of freedom; Akaike Information Criterion (AIC) = 344.18.

Relationship Between Reported Reminder Use and Thought Probe Responses

To assess whether participants' self-reported intention to use reminders predicted the proportion of future-oriented and task-related thinking during the retention interval, we conducted three multiple linear regressions. Each model included Reminder Use (1 = Yes, 0 = No), Reminder Reliability Condition (50% = reference), Session (Trust = reference), and Reminder Availability (Experiment 3 = reference) as predictors. The outcome variables were the proportion of PM-related thoughts, Task-related thoughts, and Task-unrelated thoughts, respectively.

PM-related thoughts. The regression model predicting the proportion of PM-related thoughts was significant, $F(4, 419) = 3.13$, $p = .015$. The residual standard error was 0.299 on 419 degrees of freedom. The model explained $R^2 = .03$ of the variance, with an adjusted $R^2 = .02$. Residuals ranged from -0.349 to 0.842, with the first quartile at -0.241, the median at -0.158, and the third quartile at 0.092. The intercept was significant, $\beta = 0.338$, SE = 0.046, $t = 7.40$, $p < .001$. Reminder Use was not a significant predictor, $\beta = -0.082$, SE = 0.042, $t = -1.95$, $p = .052$. Condition was significant, $\beta = -0.083$, SE = 0.029, $t = -2.86$, $p = .004$. Session was not significant, $\beta = 0.012$, SE = 0.029, $t = 0.41$, $p = .686$. Availability was not significant, $\beta = -0.014$, SE = 0.029, $t = -0.48$, $p = .630$.

Task-related thoughts. The regression model predicting the proportion of Task-related thoughts was significant, $F(4, 419) = 3.38$, $p = .010$. The residual standard error was 0.318 on 419 degrees of freedom. The model accounted for $R^2 = .03$ of the variance, with an adjusted $R^2 = .02$. Residuals ranged from -0.794 to 0.437, with the first quartile at -0.206, the median at 0.072, and the third quartile at 0.276. The intercept was significant, $\beta = 0.608$, SE = 0.049, $t = 12.49$, $p < .001$. Reminder Use was not significant, $\beta = 0.071$, SE = 0.045, $t = 1.57$, $p = .118$. Condition was significant, $\beta = 0.088$, SE = 0.031, $t = 2.86$, $p = .005$. Session was not significant, $\beta = -0.044$, SE = 0.031, $t = -1.44$, $p = .152$. Availability was not significant, $\beta = 0.028$, SE = 0.031, $t = 0.90$, $p = .369$.

Task-unrelated thoughts. The regression model predicting the proportion of Task-unrelated thoughts was not significant, $F(4, 419) = 1.41$, $p = .229$. The residual standard error was 0.160 on 419 degrees of freedom. The model explained $R^2 = .01$ of the variance, with an adjusted $R^2 = .00$. Residuals ranged from -0.099 to 0.933, with the first quartile at -0.085, the median at -0.061, and the third quartile at -0.047. The intercept was significant, $\beta = 0.055$, SE = 0.025, $t = 2.24$, $p = .026$. Reminder Use was not significant, $\beta = 0.012$, SE = 0.023, $t = 0.52$, $p = .606$. Condition was not significant, $\beta = -0.005$, SE = 0.016, $t = -0.35$, $p = .731$. Session was significant, $\beta = 0.033$, SE = 0.016, $t = 2.10$, $p = .037$. Availability was not significant, $\beta = -0.014$, SE = 0.016, $t = -0.89$, $p = .376$.

Relationship Between Internal Thought Content and PM Performance Without Offloading

We examined the Pearson correlation between mean PM retrieval and each thought probe category—PM-related, Task-related, and Task-unrelated thoughts—separately for Session 1 and Session 2 in the No Reminder condition. In Session 1 (Trust), the correlation between PM-related thoughts and PM retrieval was not significant, $r(106) = .152$, $p = .117$, 95% CI [−.038, .331]. Task-related thoughts were not significantly correlated with PM retrieval, $r(106) = -.086$, $p = .378$, 95% CI [−.270, .105]. Task-unrelated thoughts also showed no significant correlation, $r(106) = -.141$, $p = .145$, 95% CI [−.322, .049].

In Session 2 (Critical), PM-related thoughts were not significantly correlated with PM retrieval, $r(106) = .074$, $p = .448$, 95% CI [−.117, .259]. Task-related thoughts were likewise not significantly correlated with PM retrieval, $r(106) = .062$, $p = .525$, 95% CI [−.129, .248]. However, Task-unrelated thoughts were significantly negatively correlated with PM retrieval, $r(106) = -.261$, $p = .006$, 95% CI [−.429, −.076].
